# Why a major study on myocarditis risk following COVID vaccination should not influence public-health policy

**DOI:** 10.3389/fmed.2023.1126945

**Published:** 2023-03-23

**Authors:** Paul S. Bourdon, Spiro P. Pantazatos

**Affiliations:** ^1^Department of Mathematics, University of Virginia, Charlottesville, VA, United States; ^2^Department of Psychiatry, Columbia University Irving Medical Center, New York, NY, United States; ^3^Molecular Imaging and Neuropathology Division, New York State Psychiatric Institute, New York, NY, United States

**Keywords:** public-health policy, myocarditis, SARS-CoV-2, COVID-19 vaccination, Omicron variant

## 1. Introduction

The “Discussion” section of the recently published paper “Risk of Myocarditis After Sequential Doses of COVID-19 Vaccine and SARS-CoV-2 Infection by Age and Sex” (1) by Patone et al. begins,

In a population of >42 million vaccinated individuals, we report several new findings that could influence public health policy on COVID-19 vaccination. First, the risk of myocarditis is substantially higher after SARS-CoV-2 infection in unvaccinated individuals than the increase in risk observed after a first dose of ChAdOx1nCoV-19 vaccine, and a first, second, or booster dose of BNT162b2 vaccine.

Because of (i) Patone et al.'s use of an unreasonable definition of infection, (ii) flaws in the design of their study (a major one introduced after nearly all study data had been collected and analyzed), and (iii) the insignificant number of Omicron infections contributing to their study's findings, the conclusion drawn in the passage above is possibly false in general and highly likely to be false for children in the age range 12–17 as well as for males under age 40 receiving a second dose of Pfizer's BNT162b2.

## 2. Discussion

### 2.1. An unreasonable definition

Patone et al.'s study population consists of 42,842,345 residents of England, ages 13 and up, receiving at least one dose of a COVID-19 vaccine during the study period 1 December 2020 until 15 December 2021. The authors report 5,934,153 members of their study population “had SARS-CoV-2 infection before or after vaccination” [(1), “Results,” p. 743]. According to a technical article by the UK's Office of National Statistics (2), about 8.3% of the English population had been infected by the beginning of Patone et al.'s study period and about 43.2% had been infected by its end. Thus, roughly, we might expect about 34.9%, of the study population to have experienced an initial COVID-19 infection during the study period: 0.349 × 42, 842, 345 ≈ 14, 951, 978 initial infections, not 5, 934, 153. The undercount of infections is due to the use of the following definition: “…SARS-CoV-2 infection, defined as the first SARS-CoV-2-positive test in the study period” [(1), “Exposures,” p. 745]. Thus, Patone et al.'s finding that “the risk of myocarditis is substantially higher after SARS-CoV-2 infection in unvaccinated individuals than the increase in risk [after, say, any dose of Pfizer's BNT162b2]” is based on the untenable assumption that all infections occurring in their study population are associated with (reported) positive COVID-19 tests.

A total of 2,958,026 positive SARS-CoV-2 tests were reported for study-population members while they were unvaccinated [(1), “Results,” p. 745]. Assuming that the data from the ONS technical article cited above is accurate, we establish 4,685,095 as a *lower bound* on the number of *infections* among study-population members while unvaccinated—see the Supplementary material “Estimating the Number of SARS-CoV-2 Infections in Members of Patone et al.'s Study Population Before Vaccination.”

To understand the implications of using a more realistic count of SARS-CoV-2 infections occurring among members of the study population before they received an initial dose of a COVID vaccine, let's assume that the ratio of infections to positive tests, 1.58 ≈ 4, 685, 095/2, 958, 026, is similar for the four major demographic groups considered in the study: men < 40, women < 40, men ≥40, women ≥40. Now consider the data in Table 1 above (excerpted from Patone et al. (1), Table 3, p. 749) that express the risk to men under 40 of experiencing myocarditis after COVID vaccination or a positive SARS-CoV-2 test in terms of incident-rate ratios (IRRs). In the third column from the right, if the IRR 4.35, reflecting positive-test-linked incidence, is changed to $\frac{1}{1.58}\times 4.35\approx 2.75$, reflecting infection-linked incidence, then the resulting IRR falls below those for the second dose of Pfizer's BNT162b2 (3.08) and the first dose of Moderna's mRNA-1273 (3.06).

**Table 1:**
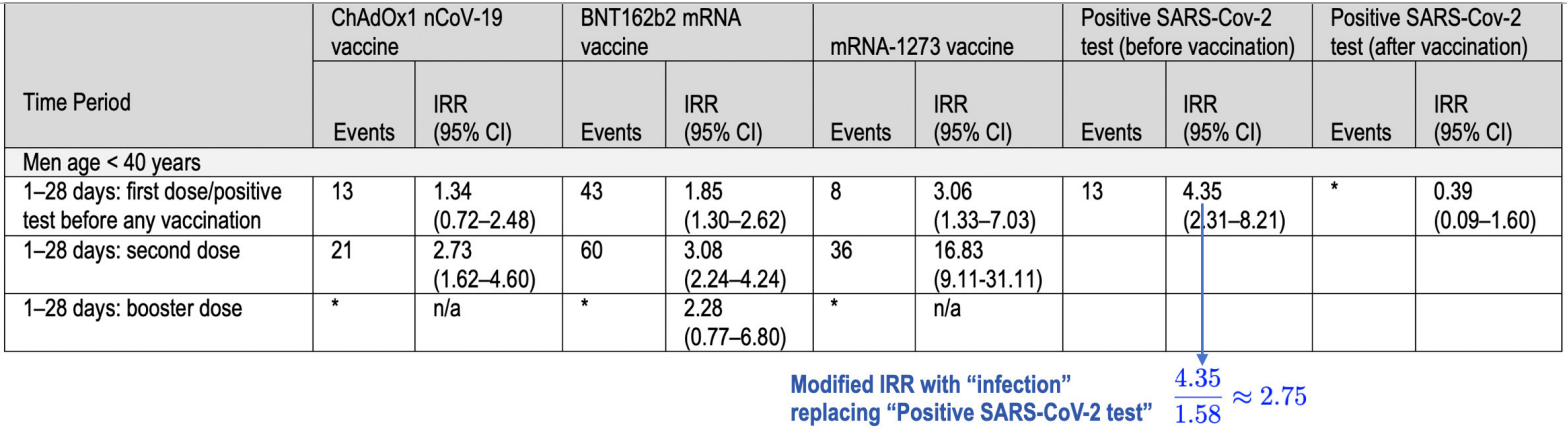
Incidence rate ratios (IRR [95% CI]) reported in Table 3 of Patone et al. (1) for myocarditis after vaccination and after a positive SARS-CoV-2 test, adjusted for calendar time from 1 December 2020 to 15 December 2021.

We are not the first to notice that Patone et al.'s study exaggerates the risk of myocarditis after SARS-CoV-2 infection. For instance, Dr. Vinay Prasad raised this issue 28 December 2021 in commenting on an earlier publication by Patone et al. based on study data from the period 1 December 2020 to 24 August 2021.^1^

### 2.2. Three design flaws

As noted in the preceding section, 2,958,026 of the study population tested positive for SARS-CoV-2 before their first vaccination; 114 myocarditis cases occurred during the study period in this subset of the population 1–28 days from the test date [(1), “Results,” p. 745]. Based on this raw data (used in Patone et al.'s “Main analysis”), the incidence of positive-test-associated myocarditis among study-population members while unvaccinated is

$Q:=\frac{114}{2958026}\approx 38.54$ cases per million positive tests per 28 days.

As we have explained, to obtain myocarditis incidence after a COVID *infection*, the denominator of the incidence quotient *Q* above must be increased to at least 4,685,095 to reflect the number of SARS-CoV-2 infections that occurred in study-population members while they were unvaccinated, yielding an incidence of 114/4, 685, 095 ≈ 24.33 per million, likely an upper bound. The numerator of *Q* is problematic as well.

For Patone et al.'s study, a case of myocarditis is one that results in death or in hospital admission for myocarditis—some of these admissions occurred in temporal proximity (1–28 days) to a COVID-19 vaccination, some in temporal proximity (1–28 days) to a positive COVID-19 test, and some, “baseline cases,” did not have either of these temporal associations.

*Flaw 1*: Because the study population consists of only vaccinated individuals and any unvaccinated person who dies from myocarditis in temporal proximity to a positive COVID test will not be able to later vaccinate, the numerator 114 won't include any cases of myocarditis resulting in death.

*Flaw 2*: COVID-related myocarditis risk among the unvaccinated is, of course, unrelated to vaccination. Because the study population consists of only vaccinated individuals, this creates an illogical dependence of Patone et al.'s computation of the incidence of positive-test-associated myocarditis among the unvaccinated on the decision to later vaccinate or not made by a very small number of individuals in England—those individuals, ages 13 and up, hospitalized with positive-test-associated myocarditis during the study period while unvaccinated. We know 114 of those individuals later chose to vaccinate, but we do not know how many chose not to vaccinate. What if none had chosen to vaccinate? Then, the numerator 114 in Patone et al.'s main analysis of incidence would be 0 and the study would have shown 0 risk of positive-test-associated myocarditis among the unvaccinated. On the other hand, if, during the study period, those (in England, age 13 and up) hospitalized while unvaccinated with positive-test-associated myocarditis later chose to vaccinate with higher probability than a “generic” unvaccinated person having had a positive COVID test, then Patone et al.'s incidence quotient *Q* will overstate risk.

Design Flaws 1 and 2, described above, were introduced into Patone et al.'s study at a late stage—after nearly all study data had been collected and analyzed. Reading the preprint version (3) of Patone et al.'s published paper (1) reveals that, as originally designed, Patone et al.'s study did not include an analysis of the incidence of positive-test-associated myocarditis among the unvaccinated. Rather, positive-test-associated myocarditis events, pre-first-dose and post-first-dose, were combined to compute myocarditis incidence following a positive test independent of vaccination status.

*Flaw 3*: Patone et al.'s description of their study in (1) doesn't include sufficient details for a reader or reviewer to determine how baseline myocarditis incidence is computed. For instance, it appears that baseline incidence depends on “seasonal variation” in myocarditis infection as well as hospital-admission pressure [(1), “Study Design and Oversight,” p. 744]; however, no details are included to explain how these factors influence baseline rates. There is evidence that myocarditis is not seasonal (4).

### 2.3. Additional limitations of study findings

Omicron-variant cases in England were first identified on 27 November 2021. By 15 December 2021, the last day of Patone et al.'s study period, the number of confirmed Omicron cases in England, totaled 10,740 (5). However, there were many unconfirmed cases. In the last section of our supplement, we show that a model developed by the UK Health Security Agency suggests that fewer than 1% of the 5,934,153 (first) positive COVID tests that contributed to the study's findings indicated Omicron infections. Clearly, Patone et al.'s risk estimates for positive-test-associated myocarditis among the unvaccinated or vaccinated do not necessarily apply to the Omicron variant, which is the variant of current public-health concern.

Omicron infection is recognized to be milder than that of previous variants. A study by Lewnard et al.(6) suggests reduced hazard ratios for severe clinical outcomes across the board for Omicron vs. Delta, with hazard reduction “starkest among individuals not previously vaccinated against COVID-19”; e,g., the adjusted hazard ratio for mortality is 0.14 (0.07, 0.28) for the unvaccinated. There is every reason to expect the infection-associated myocarditis hazard ratio—especially in the unvaccinated—is substantially reduced as well. Patone et al. do not acknowledge that their findings may not continue to be valid for the Omicron variant.

In a discussion of limitations of their study in the penultimate paragraph of their article, Patone et al. state the following:

[A]lthough we were able to include 2, 230, 058 children age 13 to 17 years in this analysis, the number of myocarditis events was small (56 events in all periods and 16 events in the 1 to 28 days after vaccination) in this subpopulation and precluded a separate evaluation of risk.

Thus, it appears there were no positive-test-associated cases of myocarditis among members of their study population in the age range 13–17. This is consistent with data in eTable 7 from a study by Karlstad et al.(7) showing 0 cases of myocarditis associated with SARS-CoV-2 infection for males and females in the age range 12–15. Thus, Patone et al.'s data, together with the data from Karlstad et al.'s study, suggests that for children between 12–17 the risk of myocarditis after vaccination is higher than that after SARS-CoV-2 infection (contrary to Patone et al.'s finding, quoted in our introduction above, suggesting the opposite is true in general).

## 3. Conclusion

We have presented ample evidence that Patone et al.'s “new findings” reported in their *Circulation* article should not influence public-health policy.

## Author contributions

PB analyzed the data and drafted the manuscript. SP edited the manuscript. Both authors contributed to the article and approved the submitted version.

## Footnotes

^1^See https://brownstone.org/articles/myocarditis-under-age-40-an-update/.
