# Supplementary material for: Why a major study on myocarditis risk following COVID vaccination should not influence public-health policy
Source: Front Med (Lausanne). 2023 Mar 23;10:1126945. doi: 10.3389/fmed.2023.1126945 (PMC10076766; doi:10.3389/fmed.2023.1126945)
Supplement: Supplementary file 1 [file Data_Sheet_1.pdf]

Supplement

1 ESTIMATING THE NUMBER OF SARS-COV-2 INFECTIONS IN MEMBERS OF PATONE ET AL.'S STUDY POPULATION BEFORE VACCINATION

Our goal is to compute a lower bound on the number of SARS-CoV-2 infections occurring during Patone et al.'s study period (1 Dec 2020 – 15 Dec 2021) in members of the study population while they were unvaccinated. Equivalently, we compute a lower bound on the number of residents of England, ages 13 and up, receiving, during the study period, a first dose of a SARS-CoV-2 vaccine after having been infected by COVID-19 during the study period. We use two sources of data to accomplish this—cumulative percentage estimates of the number of persons in England infected with SARS-CoV-2 from 30 November 2020 through 14 December 2021 from the UK's Office of National Statistics (ONS) and reports of the total number of 1st doses of COVID vaccines administered in England through a given date from the UK's National Health Service (NHS).

The UK's ONS has made publicly available a technical article "Coronavirus (COVID-19) Infection Survey technical article: Cumulative incidence of the number of people who have tested positive for COVID-19, UK" that "presents modelled estimates of the number of people who have had at least one episode of coronavirus (COVID-19) since the start of the UK Coronavirus Infection Survey (CIS) on 27 April 2020 until 11 February 2022." The modeling process entails the estimation of "the daily proportion of the population who would test positive with their first known COVID-19 infection (if they were tested)," while positive tests are used to predict infections.

Estimates of the percentage of those in the population who have had at least one episode of coronavirus infection are provided in an *Excel* spreadsheet, available for download under item 4 at the ONS webpage for the technical article described above. The data of interest are those used to create Figure 1 (for England) under item 4. Spreadsheet data are provided to seven decimal places, with display initially limited to 2 decimal places; we chose to compute our underestimate of those infected with COVID before joining the study population (via receiving an initial dose of a COVID vaccine) using the the full seven-decimal-place data in the spreadsheet. Here's a portion of the spreadsheet data.

	England		
Date	Cumulative % of the population who have ever tested positive for COVID-19	90% Lower credible interval	90% Upper credible interval
Monday, April 27, 2020	0.0710631	0.0457664	0.1075970
Tuesday, April 28, 2020	0.1201179	0.0850256	0.1685087
⋮	⋮	⋮	⋮
Monday, November 30, 2020	8.2935101	7.4529197	9.2249694
Tuesday, December 1, 2020	8.3675792	7.5205568	9.3057309
⋮	⋮	⋮	⋮
Monday, December 7, 2020	8.8724691	7.9867798	9.8509969
⋮	⋮	⋮	⋮
Sunday, December 27, 2020	12.2650742	11.1895703	13.4400962
⋮	⋮	⋮	⋮
Sunday, January 3, 2021	13.6982559	12.5369103	14.9596570
⋮	⋮	⋮	⋮
Monday, December 13, 2021	42.8599918	39.4212853	46.4849982
Tuesday, December 14, 2021	43.1801106	39.7271316	46.8191216
Wednesday, December 15, 2021	43.5315949	40.0638227	47.1856377

More accurate description

Estimated cumulative % of the population who would have tested positive for COVID-19 assuming universal testing

Table 1: *Some data from an ONS SARS-CoV-2 infection-model spreadsheet*

We see that on the first day of Patone et al.'s study period (1 December 2020), the spreadsheet provides an estimated increase of 0.0740691% in the number of persons infected by COVID in England and on the next-to-last day of the study period (14 December 2021), the corresponding increase is 0.3201188%.[1]

On the first day of the study period, 1 December 2020, we assume that all of the 42,842,345 residents of England who were to become members of Patone et al.'s study population, by receiving at least one dose of a SARS-CoV-2 vaccine, were unvaccinated. Because England's COVID vaccination program for the general public began 8 December 2020, we assume that for the first week of the study period all persons who eventually joined the study population remained unvaccinated. According to data displayed in Table 1 above, the percentage increase in the number of COVID-infected persons in England 1 December 2020 through 7 December 2020 is $(8.8724691 - 8.2935101)\% = 0.578959\%$. Thus, we estimate that during the first week of the study period $0.00578959 \times 42,842,345 \approx 248,040$ persons in the study population were infected with COVID-19 (infected persons who would later vaccinate for COVID-19).

To obtain additional estimates of those who were infected before receiving a 1st dose of a COVID vaccine, we need to track numbers of 1st vaccine doses administered during the study period. At the NHS's "COVID-19 Vaccinations Archive" page, there is a "Weekly Covid-19 vaccinations data archive." The first report available "COVID-19 weekly announced vaccinations 31 December 2020" provides the total number of persons in England receiving a 1st dose during the period between 8 December 2020 and 27 December 2020, including the endpoint dates. This is how the data is presented

Period: **All data between 8th December and 27th December**
Source: National Immunisation Management Service (NIMS)
Basis: Provider
Published: **31 December 2020**
Revised: -
Status: Published

Breakdown by age group

Time period	16-79 years old		80+ years old		All ages	
	1st dose	2nd dose	1st dose	2nd dose	1st dose	2nd dose
8-Dec to 27-Dec	261,561	-	524,439	-	786,000	-

The data is also presented in narrative form as a document in the listing "Weekly Covid-19 vaccinations statistical bulletin archive":

Overall Vaccination Activity

- In the week ending the 27th December a total of 243,039 people received an NHS vaccination for COVID-19 in England. This takes the total since vaccinations began on the 8th of December to 786,000.

The preceding makes it clearer that "between" includes the endpoint dates.

To get a lower bound on the number of persons in the study population who were COVID infected while unvaccinated during the period Dec 8–Dec 27, let's assume that throughout the Dec 8-Dec 27 interval, the

[1] Patone et al.'s study ignores events the day of a positive test owing to "small numbers" (Table-3 footnote); thus, infections occurring 15 December 2021 are irrelevant to the study.

number of unvaccinated yet to join the study population is the number on Dec 27 (midnight):

$$42,842,345 - 786,000 = 42,056,345.$$

According to the ONS-spreadsheet data appearing in Table 1, the percentage of persons infected increased from 8.8724691% on Dec 7 to 12.2650742% on Dec 27, a difference of 3.3926051%, and thus a lower bound on the number of those eventually joining the study population having contracted, during the period Dec 8–Dec 27, a COVID infection is

$$42,056,345 \times 0.033926051 \approx 1,426,806.$$

The preceding is a lower bound because during the period Dec 8–Dec 26, the number of unvaccinated yet to join the study population exceeds 42,056,345,

We continue the process described above, using weekly vaccination reports to obtain a lower bound for each week on the number of unvaccinated yet to join the study population and then multiply by the corresponding percentage increase in the infected population using ONS data illustrated in Table 1. Here's how this looks in spreadsheet form:

A	B	C	D	E
	Estimated cumulative % of the population who would have tested positive for COVID-19 assuming universal testing	Number in England who had received 1st dose by the corresponding date in Column A. Equivalently, the number of persons entering study group by date in Column A.	Number eventually in study population remaining unvaccinated as of the corresponding date in Column A. D[n] = 42845345 - C[n]	Lower-bound estimate of number of persons evetually entering the study population who were infected during period between date in Column A one row above and date in corresponding row: E[n] = D[n] x (B[n]-B[n-1]) /100
Monday,November 30, 2020	8.2935101	0 (except, say, for clinical trial participants)		
Monday, December 7, 2020	8.8724691	0 (except, say, for clinical trial participants)	42842345	248039.6122
Sunday, December 27, 2020	12.2650742	786000	42056345.00	1426805.705
Sunday, January 3, 2021	13.6982559	1092885	41749460.00	598345.6206
Sunday, January 10, 2021	14.8716695	1997304	40845041.00	479281.266
Sunday, January 17, 2021	16.0015634	3557847	39284498.00	443873.1465
Sunday, January 24, 2021	16.9130409	5792159	37050186.00	337704.1091
⋮	⋮	⋮	⋮	⋮
Sunday, November 7, 2021	37.1503717	41773322	1069023.00	9212.204145
Sunday, November 14, 2021	38.0338014	42057108	785237.00	6937.016873
Sunday, November 21, 2021	39.0026126	42215965	626380.00	6068.439595
Sunday, November 28, 2021	40.0089388	42345685	496660.00	4998.019705
Sunday, December 5, 2021	41.0848664	42486414	355931.00	3829.559866
Sunday, December 12, 2021	42.5686802	42611175	231170.00	3430.132361
Tuesday, December 14, 2021	43.1801106	42842345	0.00	0
				Lower bound estimate of number in study popuation who had an infection before vaccination is the sum of the numbers above.
				4685095.519

Table 2: *Computation of a lower bound on number infected before first dose of a SARS-CoV-2 vaccine*

Our lower-bound estimate of the number in Patone et al.'s study population who had a COVID infection during the study period before vaccination is the sum of the numbers in column E—the number on the lower right of Table 2 above, which we round down to 4,685,095.

Our justification that the estimate 4,685,095 provides a lower bound for the number of infections that occurred in members of study population during the study period before they were vaccinated is as follows:

- The computation itself, mathematically, clearly yields a lower estimate, assuming the ONS and NHS data upon which it is based is accurate.

- The computation ignores repeat infections (which should be counted in the incidence computation).
- Consider a percentage change in the infected population of say 1% over a given week W. This percentage change depends on new infections among unvaccinated persons and new infections among vaccinated persons. Given that vaccination provides some protection from infection, we would expect the number of infections among the unvaccinated during week W to increase more than 1% and among the vaccinated to increase less than 1%. Thus, using the overall percentage change of 1% to estimate the number of members of the study population who become infected during W while unvaccinated leads to an underestimate. We have used such overall percentage changes (from the ONS model) in our computation.

2 ESTIMATING FOR THE PERIOD 27 NOVEMBER 2021 THROUGH 14 DECEMBER 2021 THE NUMBER OF POSITIVE COVID-19 TESTS IN ENGLAND REFLECTING OMICRON INFECTIONS

In this section, we approximate the number of documented Omicron cases in England that occurred from 27 November 2021 through 14 December 2021.[2]

In a document titled "Methodology for estimating daily infections in England: 16 December 2021," the UK Heath Security Agency estimated that "around 24% of all COVID-19 positive cases with specimen dates on 11 December in England ... were highly likely to be the Omicron ... variant." Moreover, the Agency also estimated for the period 27 November through December 13 a constant doubling time for *infections* of 1.9 days.

For the 11th of December, the UK's Coronavirus Dashboard reports 40,517 first-episode cases for England. We assume 24% of these, 9,724, are Omicron cases. We also assume a doubling time for *cases* of 1.9 days. Thus, e.g., we would expect $9,724 \cdot 2^{1/1.9} \approx 14,005$ cases on the 12th, $9,724 \cdot 2^{2/1.9} \approx 20,171$ on the 13th, and $9,724 \cdot 2^{-1/1.9} \approx 6,752$ on the 10th. Treating December 11 as day zero, so that the 14th of December is day 3 while the 27th of November is day -14, we obtain approximately

$$\sum_{n=-14}^{3} 9724 \cdot 2^{n/1.9} \approx 94,904$$

Omicron cases through December 14th. These 94,904 cases constitute approximately 7.73% of the total number 1,227,074 of first-episode cases reported for England from 16 November 2021 through 14 December 2021 by the UK Coronavirus Dashboard.

Patone et al.'s preprint version of their *Circulation* article reports 5,185,772 positive tests among study-population members through 15 November 2021 while their *Circulation* article reports 5,934,153 positive tests, a difference of 748,381. Assuming 7.73% of these positive tests reflect Omicron infections provides an estimate of $0.0773 \cdot 748,381 \approx 57,850$ Omicron cases in the study population, and 57,850 is less than one percent of the number of first positive tests 5,934,153 of members of the study population.

[2] Recall Patone et al.'s study ignores events the day of a positive test owing to "small numbers" (Table-3 footnote); thus, positive tests reported on 15 December 2021 are irrelevant to the study.
